# DNA CpG methylation in sequential glioblastoma specimens

**DOI:** 10.1007/s00432-020-03349-w

**Published:** 2020-08-10

**Authors:** Zoltan Kraboth, Bence Galik, Marton Tompa, Bela Kajtar, Peter Urban, Attila Gyenesei, Attila Miseta, Bernadette Kalman

**Affiliations:** 1 Institute of Laboratory Medicine, School of Medicine, University of Pecs, Pecs, Hungary; 2 Szentagothai Research Center, University of Pecs, 20. Ifjusag street, Pecs, 7624 Hungary; 3 Institute of Pathology, School of Medicine, University of Pecs, Pecs, Hungary; 4 Department of Clinical Molecular Biology, Medical University of Bialystok, Białystok, Poland

**Keywords:** Glioblastoma, DNA CpG methylation, Gene ontology analyses, Tumorigenesis, Progression

## Abstract

**Purpose:**

Glioblastoma is the most aggressive form of brain tumors. A better understanding of the molecular mechanisms leading to its evolution is essential for the development of treatments more effective than the available modalities. Here, we aim to identify molecular drivers of glioblastoma development and recurrence by analyzing DNA CpG methylation patterns in sequential samples.

**Methods:**

DNA was isolated from 22 pairs of primary and recurrent formalin-fixed, paraffin-embedded glioblastoma specimens, and subjected to reduced representation bisulfite sequencing. Bioinformatic analyses were conducted to identify differentially methylated sites and pathways, and biostatistics was used to test correlations among clinical and pathological parameters.

**Results:**

Differentially methylated pathways likely involved in primary tumor development included those of neuronal differentiation, myelination, metabolic processes, synapse organization and endothelial cell proliferation, while pathways differentially active during glioblastoma recurrence involved those associated with cell processes and differentiation, immune response, Wnt regulation and catecholamine secretion and transport.

**Conclusion:**

DNA CpG methylation analyses in sequential clinical specimens revealed hypomethylation in certain pathways such as neuronal tissue development and angiogenesis likely involved in early tumor development and growth, while suggested altered regulation in catecholamine secretion and transport, Wnt expression and immune response contributing to glioblastoma recurrence. These pathways merit further investigations and may represent novel therapeutic targets.

**Electronic supplementary material:**

The online version of this article (10.1007/s00432-020-03349-w) contains supplementary material, which is available to authorized users.

## Introduction

Glioblastoma (GBM) is the most aggressive brain tumor exhibiting great variability at histopathological and molecular levels. Its development is related to the accumulation of somatic genomic rearrangements, mutations and copy number alterations (CNAs), accompanied by changes in epigenomic and gene expression profiles. In some cases, chromothripsis, a sudden catastrophic rearrangement involving one or a few chromosomes, may also play a role (Furgason et al. 2015). Numerous studies presented genomic and transcriptomic characteristics of GBM (Cancer Genome Atlas Research Network 2008; Verhaak et al. 2010; Sottoriva et al. 2013; Brennan et al. 2013; Kim et al. 2015a, b; Patel et al. 2014; Wang et al. 2016, 2017). The first comprehensive epigenomic analysis was reported by Noushmehr et al (2010), followed by several similar efforts (Nagarajan et al. 2014; Hu et al. 2016; de Souza et al. 2018; Klughammer et al. 2018). GBM is today subdivided into transcriptional and epigenomic subgroups, and the most characteristic mutational events and pathways driving its development have been identified (Cancer Genome Atlas Research Network 2008; Verhaak et al. 2010; Brennan et al. 2013; Noushmehr et al. 2010). However, most molecular analyses involved cross-sectional cohorts, since the collection of sequential samples is hindered by the aggressiveness of GBM. Nevertheless, the available sequential studies contributed invaluable information to the understanding of GBM evolution and drug resistance (Muscat et al. 2018; Erson-Omay et al. 2017).

The initial epigenetic studies determined levels of CpG methylation within the promoter of the O-6-methylguanine-DNA methyltransferase, because its silencing renders GBM more sensitive to temozolomide (Hegi et al. 2005). The first comprehensive methylome revealed the glioma CpG island methylator phenotype (G-CIMP) in correlation with the GBM proneural transcriptional subtype (Noushmehr et al. 2010).

DNA CpG methylome studies as an alternative to RNA expression profiling in FFPE GBM specimens became feasible due to the recent availability of the reduced representation bisulfite sequencing (RRBS) method. Combining RRBS with next-generation sequencing (NGS), Klughammer et al. (2018) reported the single-CpG and single allele methylation profiles, the most important pathways and inferred transcriptional subtypes of FFPE GBM specimens in the context of multidimensional clinical and molecular data.

Testing key molecular markers (Verhaak et al. 2010) by immunohistochemistry (IHC), we previously reproduced the segregation of subgroups (Nagy et al. 2019) and demonstrated the involvement of the Wnt pathways in both cross-sectional and sequential FFPE GBM (Tompa et al. 2018). To further explore mechanisms of GBM development and recurrence, here we analyzed genome-wide distribution of differentially methylated DNA CpG sites, regions and pathways in 22 pairs of sequential FFPE GBM specimens.

## Materials and methods

### Subjects of the study and samples

Surgically removed FFPE GBM specimens were obtained between 1999 and 2017, and evaluated by routine histological work up at the Institute of Pathology, University of Pecs (UP). Leftover blocks were used for these molecular analyses after receiving approval (Number: 7517 PTE 2018 and 2019) from the Regional Clinical Research Committee.

The characteristics of patients and specimens are summarized in Table 1. The diagnosis of primary (de novo) GBM was established based on standard clinical and histopathological criteria (Louis et al. 2016). After quality assessment, 24 pairs of isocitrate dehydrogenase (IDH)-1 R132H mutation negative, initial (GBM1) and recurrent (GBM2) tumor blocks were identified. Subsequently, two pairs were excluded as the patients turned out to be younger than 20 years of age, leaving 22 pairs of GBM in the study. GBM1 specimens were taken before chemoradiation, and GBM2 specimens at recurrence after chemoradiation. All but one patient received temozolomide-based chemo- and radiation therapy after the first surgery.

**Table 1:** Patients’ characteristics

Primary	RRBS ID	Recurrent	RRBS ID2	Gender	Age at onset (years)	Age at death (years)	Treatment	T1-T2 (weeks)	Overall survival (weeks)
15043	1	9849	R1	Man	50	50	No data	31	41
9501	2	3624	R2	Man	52	53	No data	33	59
15916	4	9527	R4	Woman	63	64	Surgery + irradiation	30	43
9886	5	15289	R5	Man	41	43	No data	17	70
3094	6	15302	R6	Man	59	60	Surgery + irradiation + TMZ	35	65
5526	7	13808	R7	Woman	50	52	Surgery + irradiation + TMZ	77	88
13501	8	9614	R8	Man	39		Surgery + irradiation + TMZ	40	
12732	9	17440	R9	Man	41	43	Primary: Surgery + irradiation + TMZ; Recurrent: Bevacizumab + irradiation	117	149
17578	10	7779	R10	Man	63		No data	77	
15466	11	16534	R11	Man	66		Surgery + irradiation + TMZ	56	
10379	12	7536	R12	Woman	56	61	AVAGLIO clinical study (STUPP + Bevacizumab/placebo)	199	287
14561	13	2315	R13	Man	45		AVAGLIO clinical study (STUPP + Bevacizumab/placebo)	70	
2525	14	1365	R14	Man	32	36	Surgery + TMZ, then Bevacizumab, irradiation, BCNU	177	203
14642	15	7990	R15	Man	43	46	Surgery + irradiation + TMZ	135	192
5693	16	612	R16	Woman	45	48	Surgery + irradiation + TMZ	143	169
7183	17	11956	R17	Woman	57	59	Surgery + irradiation + TMZ	51	95
6795	18	17545	R18	Woman	61	62	Surgery + irradiation + TMZ	31	54
16189	19	16742	R19	Woman	53	55	Surgery + irradiation + TMZ	55	69
8117	20	2908	R20	Woman	37	40	Surgery + irradiation + TMZ	92	106
3997	21	5120	R21	Man	62	63	Surgery + irradiation + TMZ	58	62
10776	23	2168	R23	Man	43	44	Surgery + irradiation + TMZ	29	46
13956	24	12107	R24	Man	60	62	Surgery + irradiation + TMZ	49	60

This table summarizes the gender, age at onset and age at death of patients, the treatment modalities and T1-T2 time. OS could not be calculated for four patients because the time of death was unavailable after extensive search of all electronic medical records. Therefore, instead of OS, the T1-T2 time values were used in the statistical analyses

*TMZ* temozolomide

In control group 1 (CG1), six postmortem FFPE normal brain specimens were included from the tissue archive of the Pathology Institute, UP. This unideal choice was necessitated because no surgically dissected normal brain or other neurological disease control FFPE specimens were available. In control group 2 (CG2), DNA CpG methylation data of five brain specimens obtained during epilepsy surgery were included by downloading from the EBI European genome–phenome archive (accession number: EGAS00001002538) (Klughammer et al. 2018). DNA specimens of CG1 were processed by the same methods as GBM1 and GBM2. DNA specimens of CG2 were also processed by RRBS, but sequenced on Illumina HiSeq 3000 and 4000 machines (Klughammer et al. 2018).

Evaluation of a hematoxylin–eosin stained section from each tumor block allowed us to exclude normal brain contamination, necrosis or highly vascular regions. The characteristics of the tumors are summarized in Table 2.

**Table 2:** Characteristics of tumors

GBM1	RRBS ID	MI	MVP	Necrosis	Cell	TIL
15043	1	36	Yes	None	Astrocytic	Moderate
9501	2	2	Yes	Extensive with palisade	Astrocytic	Moderate
15916	4	10	Yes	None	Astrocytic	None
9886	5	91	Yes	Extensive	Astrocytic	None
3094	6	120	Yes	Extensive	Astrocytic	Dense
5526	7	20	Yes	Extensive	Astrocytic	Sparse
13501	8	13	Yes	Extensive	Epithelioid	Sparse
12732	9	2	Yes	Extensive with palisade	Astrocytic	Moderate
17578	10	0	No	Extensive	Astrocytic	Dense
15466	11	18	Yes	Extensive	Astrocytic	Dense
10379	12	30	Yes	Extensive	Small cell	Dense
14561	13	36	Yes	Extensive with palisade	Astrocytic	Sparse
2525	14	38	Yes	Extensive with palisade	Astrocytic	Sparse
14642	15	78	Yes	Extensive with palisade	Small cell	Dense
5693	16	42	Yes	Extensive with palisade	Astrocytic	Sparse
7183	17	44	Yes	Extensive with palisade	Astrocytic	Sparse
6795	18	15	Yes	Extensive	Astrocytic	Sparse
16189	19	24	No	None	Small cell	Sparse
8117	20	25	Yes	None	Astrocytic	Dense
3997	21	12	Yes	Palisade	Astrocytic	Sparse
10776	23	32	Yes	Extensive	Astrocytic	Sparse
13956	24	32	Yes	Extensive	Astrocytic	Sparse

GBM2	RRBS ID	MI	MVP	Necrosis	Cell	TIL
9849	R1	100	Yes	Extensive with palisade	Small cell	Sparse
3624	R2	2	No	None	Astrocytic	Dense
9527	R4	32	Yes	Extensive with palisade	Small cell	None
15289	R5	94	Yes	Extensive	Astrocytic	Sparse
15302	R6	4	No	Extensive	Astrocytic	Sparse
13808	R7	21	No	None	Astrocytic	Sparse
9614	R8	20	No	Extensive with palisade	Epithelioid	Sparse
17440	R9	14	No	Extensive	Astrocytic	Sparse
7779	R10	50	Yes	Palisade	Astrocytic	Moderate
16534	R11	14	Yes	Extensive	Astrocytic	Dense
7536	R12	62	Yes	Palisade	Astrocytic	Dense
2315	R13	36	Yes	Palisade	Astrocytic	Sparse
1365	R14	40	Yes	None	Astrocytic	Dense
7990	R15	16	Yes	Extensive with palisade	Small cell	Dense
612	R16	12	Yes	Extensive with palisade	Astrocytic	Dense
11956	R17	22	Yes	Palisade	Astrocytic	Sparse
17545	R18	18	No	Extensive with palisade	Astrocytic	Sparse
16742	R19	18	No	Extensive with palisade	Small cell	Sparse
2908	R20	20	Yes	Palisade	Astrocytic	None
5120	R21	18	Yes	Extensive with palisade	Astrocytic	Sparse
2168	R23	10	No	None	Small cell	Dense
12107	R24	16	No	Palisade	Astrocytic	Dense

This table summarizes histopathological characteristics of GBM1 and GBM2. Histological parameters were assessed by manual eyeballing using low microscopic magnification (100x) and semiquantitative evaluation criteria published previously (Tompa et al. 2018). In statistical analyses, semiquantitative determinants were replaced by numerical values: e.g. TIL: no = 0, sparse = 1, moderate = 2, dense = 3

*MI* mitotic index (number of mitoses per 10 high power fields), *MVP* microvascular proliferation, *TIL* tumor infiltrating lymphocytes

### DNA isolation

Five cuts per FFPE block were used for DNA extraction with the QIAamp DNA FFPE Tissue Kit (Qiagen GbmH, Hilden, Germany). DNA quantitation was carried out using a Qubit™ 1X dsDNA HS Assay Kit on a Qubit 3 Fluorimeter (Invitrogen, Carlsbad, CA, USA). The distribution of DNA fragments was determined by an Agilent Genomic DNA ScreenTape Assay on an Agilent 4200 TapeStation System (Agilent Technologies, Santa Clara, CA, USA).

### DNA methylation profiling

Bisulfite converted libraries were prepared from DNA by using the Premium RRBS kit 24x (Diagenode SA, Seraing, Belgium) according to the manufacturer’s instructions. To compensate for higher degrees of fragmentation, we increased from the recommended 200 ng to higher amounts (350–400 ng) of input DNA. DNA digestion by Msp1 was then followed by fragment-end repair and adaptor ligation. The amount of effective library was determined using the Kapa Sybr Fast qPCR kit (Kapabiosystems, Cape Town, South Africa) on a StepOnePlus Real-Time PCR System (Applied Biosystems, Foster City, CA, USA). Samples with similar quantitative (q)PCR threshold cycle (Ct) values were multiplexed in pools of eight. The pools were subjected to bisulfite conversion, followed by a second qPCR to determine the enrichment amplification cycles for the final PCR on a GeneAmp PCR Systems 9700 (Applied Biosystems, Foster City, CA, USA). After confirming the adequate fragment size distributions on the 4200 TapeStation System and the concentrations by the Qubit 3 Fluorometer, the amplified libraries were sequenced using the NextSeq 500/550 High Output Kit v2.5 (75 cycles) on a NextSeq 550 machine (Illumina, San Diego, CA, USA). Raw sequencing data were uploaded to the European Nucleotide Archive (https://www.ebi.ac.uk/ena, Primary Accession: PRJEB38380, Secondary Accession: ERP121800). The glioma CpG island methylator phenotype (G-CIMP) was excluded with high probability in the GBM1 and GBM2 cohorts by adapting the eight gene method for bisulfite-converted sequence data (Noushmehr et al. 2010).

### Bioinformatics

After the quality control step using FastQC, sequences were filtered to remove low-quality bases and adapters by TrimGalore. Bisulfite-treated reads were aligned to the hg19 reference genome and methylation calls were performed using Bismark (Krueger and Andrews 2011). After obtaining the CpG calls, RnBeads (Müller et al. 2019) was run to identify differentially methylated sites, regions and pathways in the cohorts. The Locus Overlap Analysis (LOLA) program (in RnBeads) was used for an enrichment analysis of genomic region sets and regulatory elements (Sheffield and Bock 2016). Biological interpretation of data was assisted by the BioMethyl R package. All raw sequencing data, reports and results were stored in-house on a network-attached storage (NAS) server.

### Statistics

Patients’ age, gender and time to recurrence (T1-T2) were correlated with histological characteristics using the Kruskal–Wallis and Mann–Whitney *U* tests, and Pearson’s correlation.

## Results

### DNA CpG methylation data in FFPE control and GBM specimens

We compared DNA CpG methylation patterns in normal brain tissues and IDH-wild-type GBM specimens at initial diagnosis (GBM1) and at first recurrence (GBM2). Two control groups were initially considered. CG1 included the DNA CpG methylomes of six postmortem normal brain regions from individuals who passed away from non-neurological causes. CG2 included the DNA CpG methylomes of five FFPE brain tissues obtained during epilepsy surgery and deposited in a publicly available database (Klughammer et al. 2018). Twenty-two pairs of sequential surgically obtained FFPE GBM specimens in GBM1 and GBM2 represented the main study groups with clinical variables of age, gender, time between first and second surgery (T1–T2), OS and treatment parameters (Table 1). Pathological characteristics included tumor cell morphology, mitotic index, degree of immune infiltration and necrosis (Table 2).

TapeStation analyses revealed that DNA fragmentation was slightly, but not significantly higher in GBM1 than in GBM2 (21.65% vs. 25.10% of DNA fragments were above 2000 bp, respectively). For comparison, the DNA fragment rates above 2000 bp were 87.15% in freshly drawn total blood and 70.18% in buffy coat (Supplementary Table 1).

The mean bisulfite conversion rate that reflects the chemical conversion of unmethylated cytosine to uracil was 98.48% (Supplementary Table 2). Using the spike-in controls, the mean underconversion rate was 1.32%, and the mean overconversion rate was 1.70% (Supplementary Table 2). At an average alignment rate of 69%, the mean number of informative CpGs per sample was 20,741,979 and the median number of CpGs was 16,574,809 in the non-deduplicated raw data, representing over ten times higher than expected figures due to duplications during library amplification. Deduplication is not recommended, since it could result in biases in the CpG representation and a loss of information. Instead, 19,936 sites with overlapping SNPs were removed and CpGs with extremely high coverage were filtered out for the correction of duplicated sequences during bioinformatics preprocessing.

There was a trend for fewer informative CpGs in samples with lower quality of DNA. In CG2 (Klughammer et al. 2018), a higher mean CpG methylation rate (47.91%) was noted compared to that of CG1 (32.31%), a difference attributable to DNA quality differences in surgical and postmortem FFPE specimens. TapeStation analysis of DNA from CG1 showed the highest levels of DNA fragmentation (the mean rate of DNA fragments above 2000 bp was only 5.91%) among all groups. Based on these observations, we abandoned the CG1 data, and used the CG2 data as reference in all subsequent analyses (Klughammer et al. 2018).

Overall, a shift toward hypomethylation was observed when comparing the controls and the sequential tumor samples (the mean CpG methylation levels in CG2: 47.91%; in GBM1: 41.34%; and in GBM2: 31.6%). The methylation differences showed only a trend in the GBM1–CG2 comparison (Kruskal–Wallis test *p* = 0.35), but reached significance in the GBM2–GBM1 (*p* = 0.046) and GBM2–CG2 (*p* = 0.032) comparisons (Supplementary Table 3).

### Differential DNA methylation profiles in CG2, GBM1 and GBM2

The sample summary table in RnBeads with filtered and corrected data revealed a mean CpG site number of 60,169.48 and mean coverage of 366.07. In addition to CpG sites, four regions were covered by the analyses: tiling, genes, promoters and CpG islands (Supplementary Table 4). We primarily focused on differential methylation rates in gene promoters comparing CG2–GBM1, CG2–GBM2, and GBM1–GBM2 in all analyses.

Differential methylation on the site and region levels revealed no FDR corrected *p*-values of ≤ 0.05 in the scatterplots in the three group-wise comparisons.

In the GO analyses, hypermethylation was observed within promoters of genes related to pathways of neuronal differentiation and morphogenesis, and transcription and metabolic processes in GBM1 compared to CG2. The most significantly hypermethylated gene promoters were related to gastrulation regulation (OTX2 *p* = 8.38E-05) and cellular responses to the fibroblast growth factor (FGR) (PTBP1 *p* = 5.52E-13; POLR2D *p* = 6.09E-09; NOG *p* = 4.32E-07). There were, however, also genes showing higher degrees of promoter methylation, but with lower degrees of significance. For instance, 17 different promoters in genes associated with nucleic acid-templated transcription had hypermethylation in GBM1 compared to CG2 (mean *p* = 0.0079). Eighteen promoters in genes associated with the regulation of different nucleobase-containing compound metabolic processes were hypermethylated (mean *p* = 0.0088). Furthermore, there were 19 hypermethylated promoters of genes associated with pathways of neuron morphogenesis and differentiation in GBM1 compared to CG2.

Pathways with promoter hypomethylation in GBM1 compared to CG2 included genes related to synapse organization and assembly, neuronal ensheathment and endothelial cell proliferation. The most significantly hypomethylated pathways were also the ones in which numerically the most promoters were hypomethylated. These pathways associated with regulation of postsynapse organization (e.g., GHSR; HSPA8; FZD9; SEMA3F), synapse assembly (e.g., AMIGO1; NTRK1; THBS2), endothelial cell proliferation (e.g., HIF1A; EGFL7; TNFSF12; PRKD2) and myelination (e.g., NKX6-2; KCNJ10; NCSTN; TENM4).

The GBM2–CG2 comparison showed pathways with gene promoter hypermethylation associated with transcription regulation, cell adhesion and morphogenesis and embryonic development in the GBM2 samples. Pathways which showed the most significant hypermethylation in promoters were associated with appendage morphogenesis and limb development (e.g., ALX3; HOXD10; NOG; FRAS1; SALL4). The pathways with numerically the most promoters hypermethylated were associated with transcription regulation by RNA polymerase II and cell adhesion processes. Pathways with hypomethylated gene promoters in GBM2, compared to CG2, included a few associated with purine and pyrimidine nucleobase transports (SLC28A1), Golgi transports (SNX12; SGSM2; GCC2) and allantonin catabolic processes (ALLC).

Comparing GBM1 and GBM2, the GO analysis revealed several pathways of biological relevance. Pathways with gene promoter hypermethylation in the recurrent compared to the primary tumors included genes related to regulation of the Wnt pathway, catecholamine secretion and transport, and cellular response, signaling and communication. The most significantly hypermethylated pathways in the recurrent samples were those associated with catecholamine secretion regulation (SYT15; SYT17; PINK1; OXTR), catecholamine transport (SLC18A2; TOR1A) and signaling receptor activity regulation (CACNG8; TSG101; DLG1) as well as those negatively regulating the canonical Wnt signaling pathways (e.g., ASPM; UBAC2; KREMEN), and some Wnt ligands and receptors. However, the pathways with numerically the most hypermethylated promoters in GBM2 were those associated with regulation of the stimulus response (e.g., NDUFA13; DROSHA; FMR1), cell communication (e.g., PTP4A3; FRMPD1; PRKD2), signaling (e.g., MBIP; RNF6; NOD1) and localization (e.g., KCNJ3; KDM1A; TRIM8; PRKD2).

Pathways with promoter hypomethylation in the recurrent compared to the initial tumors included genes related to both the innate and adaptive immune responses, cellular processes and cell differentiation. The most significant *p* values were noted in pathways linked to the regulation of lymphocyte-mediated immunity (TFRC; FOXJ1; IL4R), natural killer (NK) cell-mediated cytotoxicity (e.g., HAVCR2; SERPINB9; LAMP1; CADM1) and regulation of cell killing (e.g., ICAM1; MICA; DUSP22; FERC2). In addition, several other important regulators of immune response were hypomethylated (adaptive immune response, NK- and leukocyte-mediated immunity, T cell-mediated cytotoxicity) in the recurrent samples compared to the initial GBMs. Finally, the most hypomethylated and most numerous (altogether 11) promoters, although with the least significant *p* values (mean *p* = 0.0098), were detected in cell proliferation pathways in GBM2.

### Enrichment for genomic region sets and regulatory elements

The LOLA program was run to enrich for genomic region sets and regulatory elements relevant to the interpretation of functional epigenomics data (Sheffield and Bock 2016). Here, we primarily focused on the top-ranking 1000 hyper- and hypomethylated tiling regions. In both the CG2–GBM1 and CG2–GBM2 comparisons, we identified strong enrichment in hypomethylated regions in the tumors for binding sites of transcription factors (e.g., RUNX1, ESR1, ESR2 and CTCF) and histone proteins (e.g., H3K4me1; H3K4me2; H3K4me3; H3K9me3; H3K27me3) relevant to proper embryonic stem cell differentiation and lineage fidelity maintenance. In the GBM1–GBM2 comparison, GBM2 tumors showed enrichment in binding sites for transcription factors (e.g., FOXA2, ESR1, ESR2, RXR) and histone proteins (e.g., H3K27me3, H3K9m3, H3K4me1, H3K4m2, H3K4m3) among the hypomethylated regions.

### Correlation between pathological and clinical data

We detected no association between T1–T2 and gender or the age of patients, or T1–T2 and morphological subtype, mitotic rate, microvascular proliferation or necrosis of the tumors. However, a trend for association was found between T1–T2 and the amount of tumor infiltrating lymphocytes (TIL) in the GBM1 samples (Kruskal–Wallis test *p* = 0.08), but not in the GBM2 samples (*p* = 0.737). Neither Mann–Whitney nor Pearson’s correlation analysis showed a link between TIL and mitotic rate.

## Discussion

**Fig. 1:**
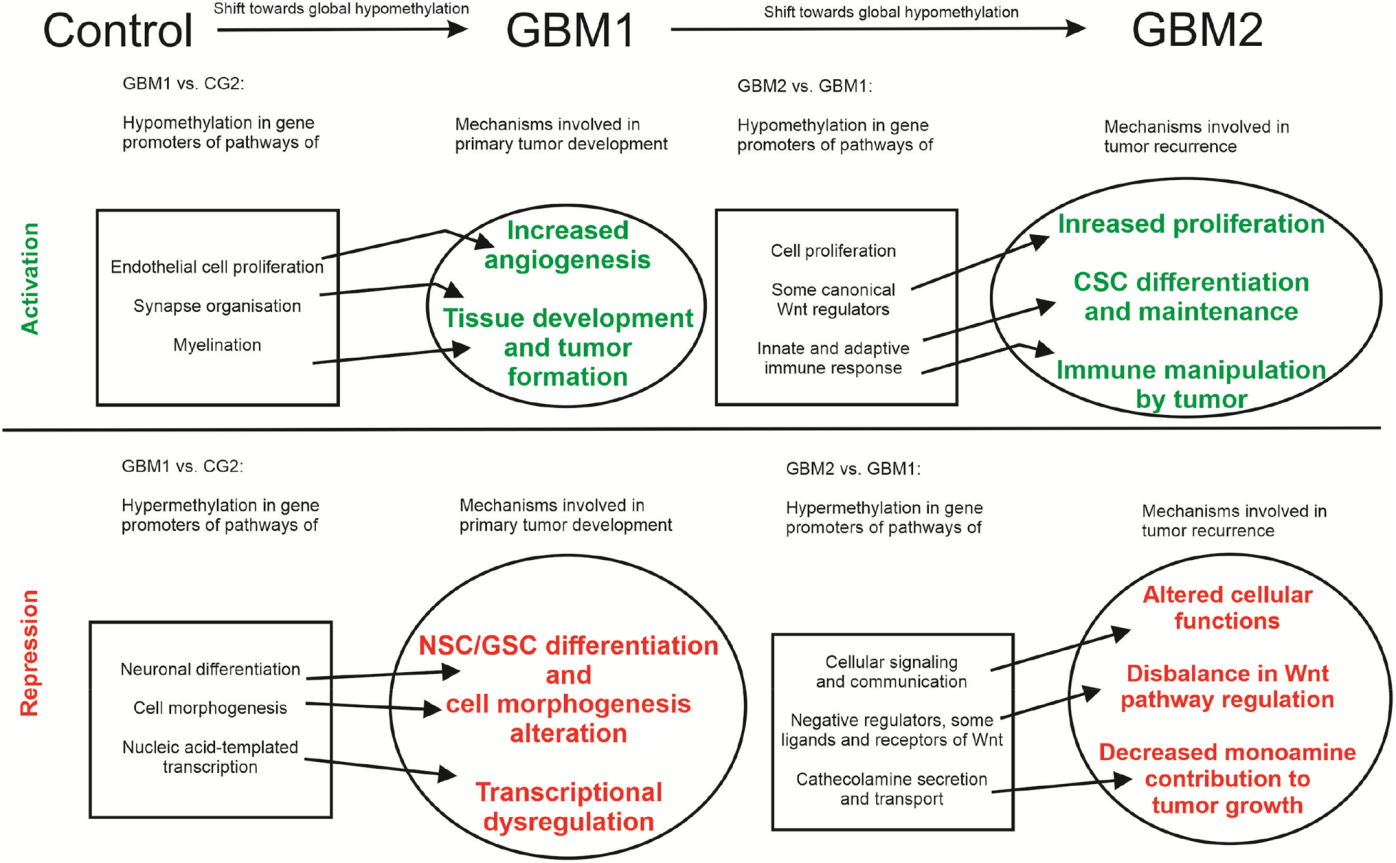
Mechanisms of GBM development and recurrence revealed by DNA CpG methylation. This figure provides a schematic depiction of molecular pathways and potential mechanisms contributing to GBM development and recurrence as revealed by RRBS and NGS of sequential GBM specimens

In this study, we aimed to identify molecular drivers and pathways essential for GBM development and recurrence (Fig. 1). We analyzed genome-wide DNA CpG methylation patterns to infer the expression of genes defining the most critical pathways in 22 paired FFPE specimens (GBM1; GBM2) from 18 years.

Although quality assessment revealed that DNA specimens from surgically removed FFPE GBM were significantly more fragmented than that of freshly obtained blood (fragments above 2000 bp in buffy coat: 70.18%; whole blood: 87.15%; GBM1: 21.65% and GBM2: 25.10%), these samples worked well in RRBS. However, because DNA quality was profoundly further compromised in the postmortem CG1 (mean fragment rate above 2000 bp: 5.91%), and the number of methylated CpG sites proportionally decreased with increasing fragmentation consistent with previous reports (Klughammer et al. 2018; Wang et al. 2013; Gillio-Meina et al. 2016), we abandoned CG1, and focused all analyses on the CG2, GBM1 and GBM2 cohorts. CG2 included DNA CpG methylomes of five brain specimens obtained during epilepsy surgery (Klughammer et al. 2018). We did realize that such DNA controls from heterogeneous populations of all normal and some degenerative cell types of adult brains may not be ideal references for the methylome from transformed glial tumor cells of GBM. However, using glial cell lines as control DNA source would be compounded by other shortcomings including oligodendroglial or astrocytic lineage specification and potential in vitro (including epigenomic) modifications. As no ideal control tissue is available for human GBM, we chose using the control brain methylomes that have been successfully applied in a similar epigenomic analysis (Klughammer et al. 2018). Using normal or near normal brain tissue as control also matches with the strategy of other GBM epigenomic studies (Noushmehr et al. 2010; Etcheverry et al. 2010).

As previously established (Nagarajan et al. 2014; Makos et al. 1992; Feinberg et al. 1988; Brothman et al. 2005; Ehrlich 2009; Hansen et al. 2011), we also observed a shift toward global DNA hypomethylation when comparing CG2, GBM1 and GBM2 (where the mean methylation rates were 47.91%, 41.34% and 31.6%, respectively).

Comparisons of differential methylation data at site and region levels revealed no significant *p* values in any of the three pairwise comparisons. GO analyses, however, highlighted several pathways with biological relevance. In the comparison of GBM1 vs. CG2, we found significant hypomethylation (likely activation) in the pathway of positive regulation of endothelial cell proliferation, a factor contributing to angioneogenesis, and thereby promoting GBM growth (Fisher et al. 2005). This finding was not surprising, as upregulation of VEGF transcripts has also been described (Etcheverry et al. 2010), and anti-angiogenetic therapy (i.e., bevacizumab) has been used to prolong progression-free survival and to reduce clinical symptom burden in GBM (Ameratunga et al. 2018; Roth et al. 2020). In the same GBM1 vs. CG2 comparison, we found hypermethylation (repression) in pathways of nucleic acid-templated transcription and different nucleobase containing metabolic processes, which affect multiple genes whose abnormal function may modify cell function and define subtype formation (Cuperlovic-Culf et al. 2012; Marziali et al. 2016). The most strikingly hypermethylated pathways were related to neuronal differentiation, while hypomethylated pathways included those related to synapse formation and myelination. We postulate that these latter findings reflect a disturbed balance in elements of a normal neuronal differentiation underlying the distorted patterns also observed by other investigators in cancer stem cells (CSCs) and in GBM (Etcheverry et al. 2010; Silvestris et al. 2019).

Comparing differential promoter methylation in GBM2 vs. CG2, the hypomethylated pathways were primarily related to intracellular function and transport, offering new targets for experimental intervention (Fallacara et al. 2019). The hypermethylated pathways included transcriptional regulation, cell adhesion and embryonic development, which may also contribute to a distortion of normal neuronal differentiation and abnormal proliferation of pluripotent neuroepithelial cells, thereby defining progression of GBM (Bradshaw et al. 2016a, b; Etcheverry et al. 2010).

The comparison of differentially methylated pathways in GBM2 vs. GBM1 revealed a number of changes involving essential cellular functions that may contribute to GBM progression. Higher gene expression and activity were inferred from the lower methylation of elements essential in cell response, signaling and communication in GBM1 than in GBM2. Elements of the canonical Wnt signaling pathway, particularly those regulating endothelial cell migration, cell adhesion or wound healing also appeared more active in GBM1 compared to GBM2. However, elements of this pathway included KREMEN1 that is capable of blocking Wnt signaling, ASPM that is essential for normal mitotic spindle function to regulate neurogenesis, or UBAC2 that has a role in degradation of Wnt receptor FZD6 and LRP6 to negatively regulate the canonical Wnt signaling pathway. Further, certain ligands (e.g., Wnt11) and receptors (e.g., Fzd8) were also significantly less methylated in GBM1 as compared to GBM2. In contrast, promoters of other Wnt ligands (e.g., Wnt6, Wnt7b) and receptors (e.g., Fzd1, Fzd3) were less methylated in GBM2 than in GBM1. Although not in sequential samples, but in GBM and control brain comparisons, a differential methylation of Wnt genes (e.g., Wnt2, Fzd6) and pathways, and both up- (Wnt5a, Fzd7, Fzd5) and downregulation of Wnt pathway transcripts (Wnt10b, Wnt7a, Wnt7b, Wnt2b) have been noted (Etcheverry et al. 2010; Nagarajan et al. 2014). In a similar DNA CpG methylation and biological pathway analysis in sequential GBM, Klughammer et al (2018) found Wnt pathway genes among those whose promoters lost methylation over time. Altogether, our data, overlapping with results in other publications, showed that negative regulators of the Wnt pathway are more active in GBM1 than in GBM2, while changes in methylation patterns occur in both directions for ligands and receptors, suggesting that the shifts and balances in Wnt pathway elements are complex during evolution of GBM. As the canonical Wnt pathway is involved in CSC stemness, tumor invasiveness and angiogenesis, our finding likely points to an important determinant of GBM evolution (Etcheverry et al. 2010; Klughammer et al. 2018; Tompa et al. 2018; Hu et al. 2016; Mazieres et al. 2005; Lamb et al. 2013; Anastas and Moon 2013). In line with this conclusion, numerous experimental therapies targeting Wnt pathway elements are already under investigation (Tompa et al. 2018; Zuccarini et al. 2018).

Another noteworthy result of GO analyses was the lower methylation of promoters (and thus higher inferred activity) in genes defining catecholamine secretion and transport in GBM1 compared to GBM2. Monoamine signaling in glioma initiating cells participates in hijacking normal developmental mechanisms and promotes tumor development. Synaptic monoamines in the GBM microenvironment influence tumor growth and angiogenesis (Caragher et al. 2018, 2019). The observation that these pathways are more hypomethylated (and likely more active) in GBM1 than in GBM2 reflects the biological characteristics of early- versus late-stage tumors, which merits further explorations. Differential methylation in catecholamine-encoding genes (e.g., ADRA2c, ADRA1a, DRD5, DRD2) and neurotransmitter pathways as well as differential expressions of such genes (e.g., ADRA1b, ADRA2a, ADRA2C, DRD5, DRD1) have been reported (Etcheverry et al. 2010; Nagarajan et al. 2014). The therapeutic potential of monoamines and their receptors in GBM have also been the subject of recent research studies (Caragher et al. 2018, 2019).

In contrast, GBM2 compared to GBM1 showed less methylation (and thus inferred higher activity) in immune pathway genes regulating leukocyte, lymphocyte and NK cell-mediated immunity. However, there were other immune-regulatory processes such as macrophage inflammatory protein production and CD8 + T cell proliferation that appeared more active (with promoters more hypomethylated) in GBM1 than in GBM2. These data are in consensus with the detected association between TIL in GBM1 and T1-T2, and align with previous observations concerning differential activity of various immune pathways during the development and progression of GBM (Etcheverry et al. 2010; Klughammer et al. 2018; Greaves et al. 2012; Wang et al. 2017). While the use of immune therapies has not been as efficient in GBM as in other solid tumors, there are several newer modalities with better blood–brain barrier penetrance and more robust cytotoxicity (e.g., vaccines, monoclonal antibodies, engineered T cells and stem cells, immune checkpoint inhibitors, proteasome inhibitors, RNA-based therapies, oncolytic viruses), which are expected to overcome immune evasion and to specifically target tumor cells or their microenvironment (Giotta Lucifero et al. 2020; Roth et al. 2020).

Finally, we also found support to our observations by comparing the array-based DNA CpG methylation data of TCGA GBMs to the sequence-based methylomes of our CG2 controls (Klughammer et al. 2018), and the array-based methylation data of the available 12 sequential TCGA GBM pairs to each other (https://portal.gdc.cancer.gov/repository?filters=%7B%22op%22%3A%22and%22%2C%22content%22%3A%5B%7B%22op%22%3A%22in%22%2C%22content%22%3A%7B%22field%22%3A%22cases.project.project_id%22%2C%22value%22%3A%5B%22TCGA-GBM%22%5D%7D%7D%2C%7B%22op%22%3A%22in%22%2C%22content%22%3A%7B%22field%22%3A%22files.data_category%22%2C%22value%22%3A%5B%22DNA%20Methylation%22%5D%7D%7D%5D%7D&searchTableTab=cases). The results of the first (cross-platform) analysis revealed that promoters in genes of pathways involved in embryonic development, immune regulation and Wnt signaling were less methylated (presumably more active) in the TCGA GBM samples than in the CG2 controls. The results of the second analysis showed less methylated (presumably more active) promoters in genes of pathways involved in stem cell proliferation and cell dedifferentiation, intracellular regulatory and metabolic processes, negative regulation of apoptosis, cell adhesion and T cell polarity as well as migration in the TCGA recurrent compared to the primary samples. In contrast, promoters of genes in pathways involved in endothelial cell proliferation, negative regulation of the execution phase of apoptosis, T cell proliferation, cell–cell signaling, neuronal differentiation, and regulation of G protein-mediated signaling (including neurotransmitter, catecholamine and some Wnt receptor signaling, though with lower ranking in the list) were less methylated (presumably more active) in the TCGA primary than in the recurrent samples. Considering the technical limitations and interpretive difficulties when comparing data from various platforms and also results from small cohorts, the outcome of the TCGA sample analyses in overlap with ours lends further support to our conclusions.

Enrichment analyses highlighted regions representing hypomethylated binding sites for transcription factors (e.g., ESR1, ESR2, CTCF, RUNX1) and histone proteins (e.g., H3K27me3; H3K4me1; H3K4me3) likely relevant to CSC differentiation and GBM development (Klughammer et al. 2018; Huang et al. 2004; Bernstein et al. 2006; Hyun et al. 2017). These analyses also showed an enrichment for hypomethylated binding sites of transcription factors (e.g., ESR1, ESR2, FOXA2) and histone proteins (e.g., H3K4m1, H3K4m2, H3K4m3, H3K27m3, H3K9m3) in GBM2 compared to GBM1, suggesting a role for these factors in tumor progression (Kondo et al. 2004; Steward et al. 2006).

Altogether, these methylome analyses revealed important molecular pathways and mechanisms contributing to the occurrence and progression of GBM (Fig. 1). While our methodological approach was similar to that of Klughammer et al. (2018), the presence of several modifying factors including the heterogeneous tumor biology, differences in cohorts’ sizes, distributions of patients’ age, gender and ethnic background, and the reduced representation of methylome itself may explain the partial (although still notable) overlap between the two studies. A weakness of our analyses is the omission of the full IDH1/IDH2 mutational status evaluation due to the limited availability of the archived samples, though we excluded with high probability the presence of G-CIMP. These issues, however, were not among the original aims and the information would not have modified the outcome. Also, due to the limited availability of paired primary and recurrent GBM samples, a hierarchical cluster analysis, testing for intra- and inter-tumor heterogeneity or correlations of the methylome data with other somatic molecular changes statistically would not have been meaningful. Finally, it is also important to note that gene expression regulation is a complex process involving multiple mechanisms (e.g., gene copy number variation, transcription factor expression, histone modification, microRNA and long non-coding RNA expression, or splicing), explaining that DNA CpG methylation alone may not always correlate with gene expression, and cautioning us when inferring gene transcription from promoter methylation status (Etcheverry et al. 2010). Our sequential methylome analyses in FFPE clinical specimens is, however, one of the few longitudinal GBM studies, which not only extends existing data by confirmatory information, but also identifies new elements and pathways of tumor development. Even among longitudinal analyses, this differential methylation profiling represents one of a few similar studies (Klughammer et al. 2018) and aligns with the goals of the recently formed Glioma Longitudinal Analysis Consortium (GLASS) (The Glass Consortium 2018; Barthel et al. 2019).

## Conclusions

This study in sequential FFPE tumor specimens revealed several important mechanisms that may underlie the development and progression of GBM. Pathways involved in synapse formation, myelination and endothelial cell proliferation were more active in GBM1 than in CG2, likely underlying a faulty tissue formation and angioneogenesis during tumorigenesis. The repression of elements of normal neurogenesis also might support a distorted stem cell differentiation in GBM. Pathways of basic cell response, signaling and communication as well as catecholamine signaling appeared more active in early than late phases of GBM. The inferred involvement of the canonical Wnt pathway regulation, while essential, also appeared complex regarding the development and progression of GBM. Similarly, various elements of immune regulatory pathways seemed to be differentially active in early and late stages of GBM. Altogether, this study revealed several differentially methylated pathways in GBM, which translate into differentially active genes and pathways with potential importance in new treatment development.

## Electronic supplementary material

Below is the link to the electronic supplementary material.

Supplementary Table 1. Sample fragmentation data. This table shows DNA fragment distribution in GBM1, GBM2, freshly drawn blood samples and CG1. Supplementary Table 2. Conversion efficiency data. This table summarizes conversion efficiency of bisulfite treatment in GBM1 and GBM2. Supplementary Table 3a. Methylation data of paired GBM samples (GBM1 and GBM2). Supplementary Table 3b. Methylation data of postmortem control samples (CG1). These tables show DNA CpG methylation data in GBM1, GBM2 and CG1. Supplementary Table 4. Sample coverage data. This table summarizes NGS coverage data in paired GBM samples (XLSX 41 kb)

## Data Availability

Raw sequencing data were uploaded to the European Nucleotide Archive (https://www.ebi.ac.uk/ena, Primary Accession: PRJEB38380, Secondary Accession: ERP121800). Some of the data are also provided in the Electronic Supplementary Material.

## Code availability

Publicly available bioinformatics programs and tools were used as detailed in the manuscript.
